# Phase Angle: Could Be an Easy Tool to Detect Low-Grade Systemic Inflammation in Adults Affected by Prader–Willi Syndrome?

**DOI:** 10.3390/nu12072065

**Published:** 2020-07-11

**Authors:** Luigi Barrea, Gabriella Pugliese, Giulia de Alteriis, Annamaria Colao, Silvia Savastano, Giovanna Muscogiuri

**Affiliations:** 1 Dipartimento di Medicina Clinica e Chirurgia, Unit of Endocrinology, Federico II University Medical School of Naples, Via Sergio Pansini 5, 80131 Naples, Italy; robiniapugliese@gmail.com (G.P.); dealteriisgiulia@gmail.com (G.d.A.); colao@unina.it (A.C.); sisavast@unina.it (S.S.); giovanna.muscogiuri@gmail.com (G.M.); 2 Centro Italiano per la cura e il Benessere del paziente con Obesità (C.I.B.O), Department of Clinical Medicine and Surgery, Endocrinology Unit, University Medical School of Naples, Via Sergio Pansini 5, 80131 Naples, Italy; 3 Cattedra Unesco “Educazione alla Salute e allo Sviluppo Sostenibile”, Federico II University Medical School of Naples, Via Sergio Pansini 5, 80131 Naples, Italy

**Keywords:** Prader–Willi syndrome, phase angle, obesity, chronic low-grade inflammation, nutritionist

## Abstract

Prader–Willi syndrome (PWS) is the most common genetic inherited obesity syndrome. Obesity-related complications, mostly related to chronic low-grade systemic inflammation (LGI), are the commonest cause of mortality and morbidity in PWS adults. Phase angle (PhA) is an easy tool to screen a state of LGI in healthy subjects and in subjects with obesity and is obtained from bioelectrical impedance analysis (BIA). The aim of this study was to validate the PhA in PWS adults as a potential biomarker of LGI. In this single-center, cross-sectional study, fifteen PWS adults (six males, aged 19–41 years, and body mass index (BMI) 31.0–68.0 Kg/m^2^) and fifteen control subjects matched by gender, age, and BMI were evaluated. PhA values were significantly lower (*p* < 0.001), while high-sensitivity C-reactive protein (hs-CRP) levels were significantly higher (*p* < 0.001) in PWS adults compared with controls (*p* < 0.001), without a gender difference in the latter. After adjustment for gender, BMI, and waist circumference, significant correlation was found between PhA and hs-CRP levels (r = −0.69, *p* = 0.01). At the ROC analysis, the threshold value of PhA predicting the highest hs-CRP levels above the median value was found at PhA ≤ 4.8° (*p* = 0.01; AUC, 0.82; standard error, 0.12; 95% CI, 0.58 to 1.00). These results suggest that PWS adults had a significant higher degree of LGI compared with their counterparts. Moreover, our finding suggest that PhA is a valid biomarker of LGI also in PWS adults.

## 1. Introduction

Prader–Willi syndrome (PWS) is the most common genetic inherited obesity syndrome, due to an imprinting disorder resulting from the loss of paternally derived alleles located on 15q11–q13. PWS prevalence is approximately one in 10,000 to 30,000 births without gender-related differences in incidence [1,2]. The PWS subjects presented at birth hypotonia, feeding difficulties, and retarded psychomotor development, with obsessive-compulsive characteristics and hyperphagia starting in early childhood. These patients develop during childhood a severe obesity, which is combined with multiple endocrine and metabolic disturbances, such as hypogonadotropic hypogonadism and growth hormone deficiency [3]. All these clinical conditions lead to a serious deterioration of the quality of life in adulthood [1]. The most important medical approaches to improve the pathophysiological characteristics of PWS are represented by restricting total energy intake and promoting physical activity and growth hormone replacement therapy as soon as possible [1]. Despite these medical strategies, the excess body weight represents a significant health issue in both young and adults with PWS and it is considered the main cause of morbidity and mortality in these subjects [1]. Of interest, very recently, Pacoricona Alfaro DL et al. assessed the causes of death (including cardiovascular, severe infection, respiratory, gastrointestinal, sudden death, and other causes) in French patients with PWS (both children and adults) from 2004 to 2014. Over these 11 years of study, the death toll was 104 patients with a median age at death of 30 years. PWS patients under 2 years accounted for 70% of deaths, and 17 deaths occurred in PWS patients greater than 18 years old. No gender difference was reported for both cause and age of death. More than 50% of the deaths in PWS patients (both children and adults) were due to respiratory causes [4].

Obesity, mainly visceral obesity, is well established for causing chronic low-grade systemic inflammation (LGI) (also noted as meta-inflammation). Meta-inflammation is characterized by infiltration and activation of pro-inflammatory macrophages that produce and secrete a variety of pro-inflammatory cytokines and chemokines, including high-sensitivity C-reactive protein (hs-CRP) levels [5,6], which contribute to the initiation and progression of several metabolic disorders, including cardiovascular diseases [6].

Some studies reported that PWS patients, compared with subjects without PWS, presented higher hs-CRP levels, widely recognized as a marker of LGI and a predictor of obesity and cardiovascular disease [6,7,8,9]. In PWS patients, it is often difficult to perform blood sampling due to their poor compliance, thus making the diagnosis of LGI based on biochemical parameters not always possible.

In healthy subjects and subjects with obesity, phase angle (PhA), a parameter obtained from bioelectrical impedance analysis (BIA), has been validated as an easy tool to detect LGI [10,11,12]. Indeed, PhA is equal to the arctangent of the ratio of net reactive current to the resistive current obtained by BIA instruments. PhA can be considered an excellent indicator of the physical state and cellular integrity as well as of the water distribution between the intracellular (ICW) and extracellular water (ECW) compartments [13,14]. It is well known the significant association between cellular health and inflammation [15], thereby higher PhA values suggest a greater quantity of intact cell membranes, whereas smaller PhA suggests cell death or decreased cell integrity [16]. Consequently, the PhA represents an important prognostic index for monitoring the presence and evolution of chronic inflammatory processes [17], including obesity [18,19]. 

Nevertheless, to the best of our knowledge, no studies to date have assessed PhA as potential marker of LGI in PWS adults. Therefore, the aim of this study was to evaluate if PhA could be used as screening tool of LGI also in the context of PWS, validating it by already validated inflammation marker (hs-CRP levels). 

## 2. Material and Methods

This was a cross-sectional observational, single-center study performed from October 2016 to January 2020 at the Department of Clinical Medicine and Surgery (*Endocrinology and Metabolic Diseases Unit*) Federico II Medical School, University of Naples (Italy). The study was carried out according to the Code of Ethics of the World Medical Association (Declaration of Helsinki) regarding human experimentation. The local Ethical Committee had previously approved the protocol of study (n. 173/16). After a thorough explanation of the protocol, every enrolled subject provided an informed consent. The written informed consent of participants, who were not capable to understand and give their written informed consent, was provided and signed by their legal guardian.

Recruitment strategies included 30 individuals, 15 PWS adults and 15 non-PWS adults. In particular, patients had a genetically confirmed diagnosis of PWS by a positive methylation test after attending the Unit of Endocrinology and Metabolic Diseases of the same Department. Control subjects were Caucasian volunteers with obesity coming from the same geographical area around Naples metropolitan area, Campania, Italy. In both groups, all female individuals were assessed between the third and fifth day of the menstrual cycle (in the early follicular phase), while participants of the control group reported to be not pregnant or breastfeeding. All PWS patients in our cohort were previously treated with recombinant human growth hormone treatment during childhood, as previously reported [20], having stopped treatment at least 2 years before the start of the study. The following exclusion criteria have been applied in order to make the sample more homogeneous:

- Age <18 years and >45 years;
- Smokers;
- Subjects who habitually practiced physical activity, defined as an aerobic exercise lasting at least 30 min/day;
- Presence of type 2 diabetes mellitus according to the American Diabetes Association criteria or use of hypoglycemic drugs;
- Individuals with implanted devices such as pacemakers or defibrillators, because of the theoretical possible interference;
- Current therapy with anti-obesity drugs;
- Chronic diseases that could interfere with fluid homeostasis (chronic inflammatory diseases, liver or renal chronic diseases) based on a complete medical examination and laboratory investigations.

The power sample was estimated by the differences of means ± standard deviations (SD) of PhA in PWS adults and controls (4.5 ± 0.8 vs. 5.6 ± 0.3, *p* < 0.001). When considering the number of cases needed in each group was seven, the group size was set at 15, there was a type I (alpha) error of 0.05 (95%), and a type II (beta) of 0.05, and the calculated power size was 95%. The calculation of sample size and power were performed while using Sample Size Calculator Clinical Calc [21].

The anthropometric parameters were assessed wearing light clothes and without shoes by the same nutritionist according to the International Society for the Advancement of Kinanthropometry of 2006 (ISAK 2006) [22,23,24]. Body mass index (BMI) was calculated using the following formula: weight (kg) divided by height squared (m^2^), kg/m^2^. A wall-mounted stadiometer (Seca 711; Seca, Hamburg, Germany) was used to measure height, while a calibrated balance beam scale (Seca 711; Seca, Hamburg, Germany) was used to assess weight. According to World Health Organization’s criteria, the degrees of obesity were established [25]. Using a nonstretchable measuring tape, the same nutritionist evaluated waist circumference [26]. 

As previously reported [27,28,29], the very same nutritionist performed the BIA according the European Society of Parental and Enteral Nutrition (ESPEN) [14], placing electrodes on the hand and the ipsilateral foot, according to Kushner [30], and using a BIA (101 RJL, Akern Bioresearch, Florence, Italy) phase-sensitive system (an 800 µA current at a single frequency of 50 kilohertz) [31]. The fat mass was evaluated through the prediction equation in PWS adults developed by Bedogni et al. [32,33] for female patients and by Gray et al. [34] for male patients, as also previously reported by Lazzer et al. [35]. 

More information on anthropometric measurements and body composition determination can be found in the Supplementary Materials. 

Serum hs-CRP levels were analyzed with Siemens Healthcare Diagnostics (Marburg, Germany) with a nephelometric assay with CardioPhase high sensitivity. The intra- and inter-assay coefficients of variation were <7%.

### Statistical Analysis

The data distribution was evaluated by Kolmogorov–Smirnov test, and the abnormal data (age, weight, waist circumference, resistance (R), reactance (Xc), PhA, total body water (TBW), ICW, ECW, fat mass (FM)), were normalized by logarithm. Skewed variables were back-transformed for presentation in tables and figures. Results are expressed as mean ± SD, and categorical variables are expressed as a percentage. Differences between PWS adults and controls (Weight, R, Xc, PhA, TBW, ICW, ECW, FM, fat-free mass (FFM), and hs-CRP levels) were analyzed by Student’s paired *t*-test. Gender differences in PhA between PWS adults and control group were analyzed by Student’s independent *t*-test. The correlations among PhA, age, BMI, waist circumference, and BIA parameters were assessed, and the Pearson *r* correlation coefficients were estimated. In addition, a partial Pearson’s correlation analysis was carried out after adjusting for gender, BMI, and waist circumference as potential confounding factors. A multiple regression analysis model (stepwise method), expressed as R^2^, Beta (β), and *t*, with PhA as dependent variable was used to estimate the predictive value of hs-CRP levels, Xc, ICW, ECW, ECW/ICW ratio, and FFM. Receiver operator characteristic (ROC) curve analysis was carried out in order to identify sensitivity and specificity, area under the curve (AUC), and confidence interval (CI), as well as cut-off value of PhA in detecting the lowest decrease in hs-CRP levels. Test AUC for ROC analysis was also calculated, and we entered 0.88 for AUC ROC and 0.5 for null hypothesis values. An Alfa α level of 0.05 (type 1 error) and a β level of 0.2 (type II error) were used as the cut-off values for statistical significance. Variables with a variance inflation factor >10 were excluded in order to avoid multicollinearity. Values ≤5% were considered statistically significant. Data were collected and analyzed using the MedCalc^®^ package (Version 12.3.0 1993–2012, Mariakerke, Belgium). 

## 3. Results

This cross-sectional, observational, single-center study evaluated 30 individuals. Fifteen PWS adults and fifteen controls were matched for gender, age, and BMI. In particular, in PWS adults and control group, nine (60%) subjects were females, age was 28 ± 6.8 vs. 30 ± 6.9 years, *p* = 0.66; and BMI was 43.8 ± 10.7 vs. 43.9 ± 8.8 kg/m^2^, *p* = 0.21. No differences were evident in the two groups for waist circumference (123.4 ± 27.8 vs. 112.6 ± 18.6 cm, *p* = 0.25). Weight and body composition parameters of the study population evaluated by BIA are showed in Table 1. Significant higher values of ECW/ICW ratio were found in PWS adults compared with controls (*p* < 0.001), while lower values of Xc, and PhA, were detected in PWS adults compared with control group.

**Figure 1:**
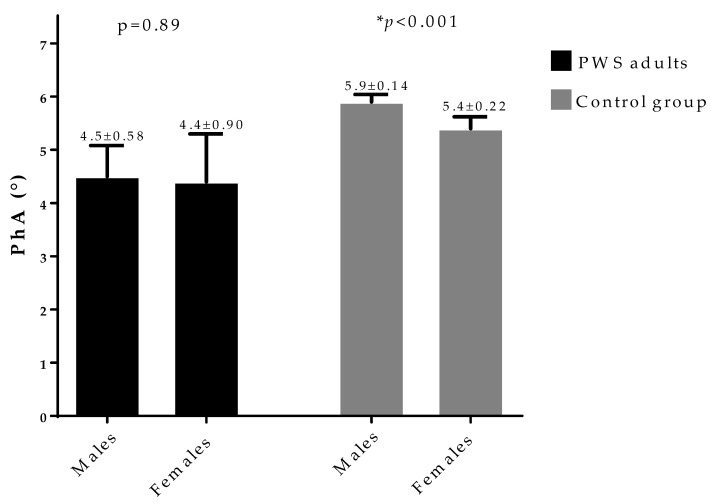
Difference in PhA in PWS adults and control group, according to gender. A clear gender difference in PhA was evident only in the control group, with higher PhA in males compared with females (*p* < 0.001). PWS, Prader–Willi syndrome; PhA, phase angle. * A *p-*value < 0.05 means a significant difference.

Figure 1 shows PhA in PWS adults and control group, according to gender. A clear gender difference in PhA was evident only in the control group, with higher PhA in males compared with females (*p* < 0.001).

Stratifying the study population by gender, no statistically significant difference was evident in fat mass among male PWS adults and control (53.9 ± 33.6 vs. 73.3 ± 23.9, *p* = 0.24) and female PWS adults and control (47.2 ± 17.7 vs. 53.8 ± 19.2, *p* = 0.40). 

**Figure 2:**
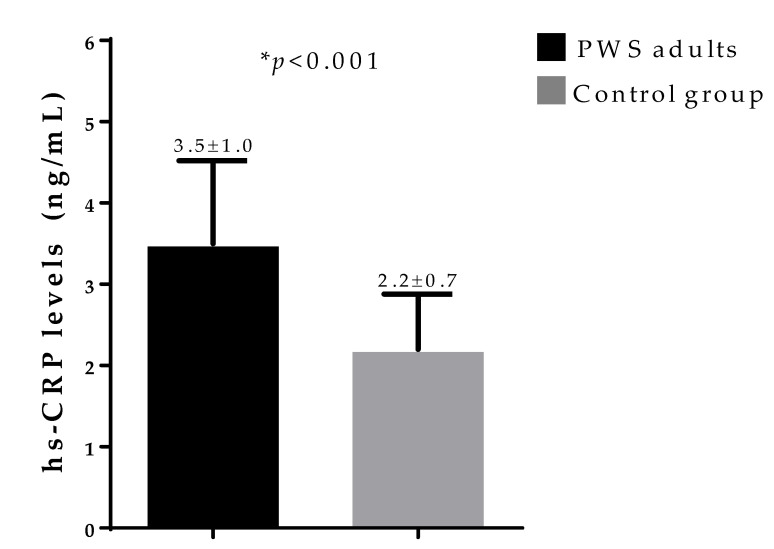
Difference in hs-CRP levels in PWS adults and control group. PWS adults presented higher hs-CRP levels compared with non-PWS adult counterparts (*p* < 0.001). PWS, Prader–Willi syndrome; hs-CRP, high-sensitivity C-reactive protein. * A *p* value <0.05 means a significant difference.

PWS adults presented higher hs-CRP levels compared with non-PWS adult counterparts (*p* < 0.001), as reported in Figure 2. 

### Correlation Analysis

Simple correlations and correlations adjusted for gender, BMI, and waist circumference among PhA and hs-CRP levels with age and anthropometric measurements in PWS adults are reported in Table 2. After adjustment for covariates, significant correlations were found between PhA and hs-CRP levels with ICW, and ECW/ICW ratio. In addition, PhA was also negatively associated with hs-CRP levels (r = −0.71, *p* = 0.003). The same correlation analyses were carried out in control group and are reported in supplementary Table S1.

**Figure 3:**
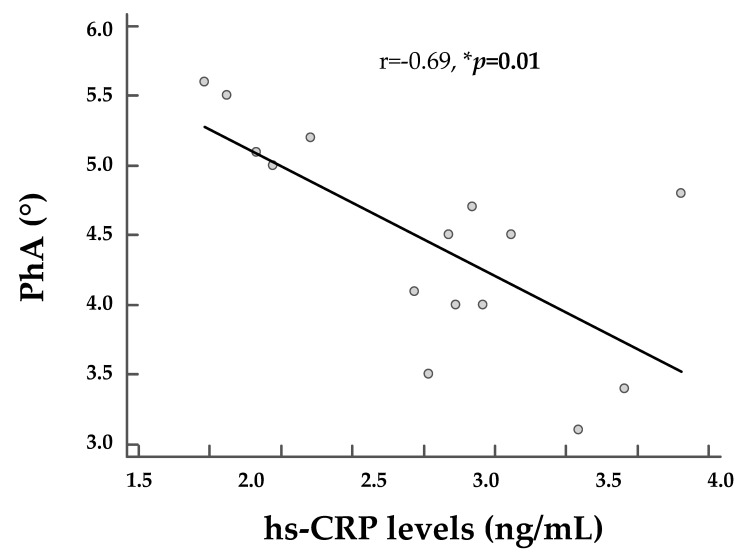
The correlation between PhA and hs-CRP levels, after adjustment for gender, BMI, and waist circumference in PWS adults. PhA showed also a negative association with hs-CRP levels (r = −0.71, *p* = 0.003), and as shown in the figure, this negative association remained also after adjustment for covariates (*p* = 0.01). PhA, phase angle; hs-CRP, high-sensitivity C-reactive protein. * A *p-*value < 0.05 means a significant difference.

Figure 3 reports the correlation between PhA and hs-CRP levels, after adjustment for gender, BMI, and waist circumference. This negative association remained also after adjustment for covariates (*p* = 0.01). Similarly, even in the control group, PhA was negatively associated with hs-CRP levels (r = −0.58, *p* = 0.02), but unlike PWS adults, this correlation was lost after adjustment for covariates (r = −0.28, *p* = 0.38). 

To compare the relative predictive power of hs-CRP levels, BMI, waist circumference, and BIA parameters associated with the PhA in PWS adults, we performed a multiple regression analysis using a model that included hs-CRP levels, BMI, waist circumference, ECW, and ECW/ICW ratio. Using this model, ECW/ICW ratio was entered at the first step (*p* < 0.001), followed by hs-CRP levels (*p* = 0.02). BMI, waist circumference, and ECW were excluded from the analysis. Results are reported in Table 3. 

**Figure 4:**
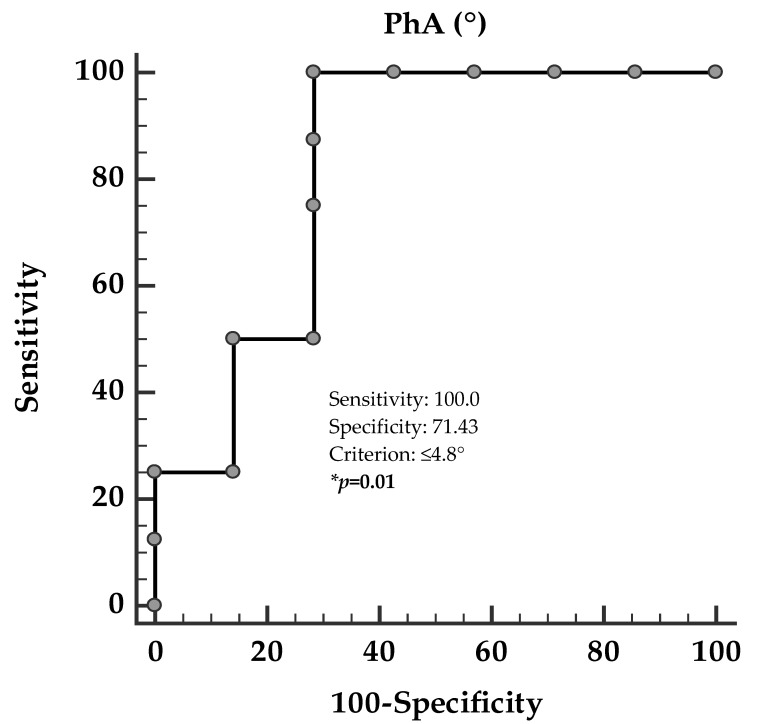
ROC for value of PhA predictive of the highest hs-CRP levels above the median value (3.68 ng/mL). At the ROC analysis, the threshold value of PhA predicting the highest hs-CRP levels (above the median value 3.68 ng/mL) was found at PhA ≤4.8° (*p* = 0.01; AUC, 0.82; standard error, 0.12; 95% CI, 0.58 to 1.00). PhA, phase angle; hs-CRP, high-sensitivity C-reactive protein. * A *p-*value in bold type denotes a significant difference (*p* < 0.05).

At the ROC analysis, the threshold value of PhA predicting the highest hs-CRP levels above the median value (3.68 ng/mL) was found at PhA ≤ 4.8° (*p* = 0.01; AUC, 0.82; standard error, 0.12; 95% CI, 0.58 to 1.00; Figure 4) in PWS adults. 

## 4. Discussion

The main finding of this research is the negative correlation between PhA and hs-CRP levels in PWS adults which allows nutritionists and clinicians to evaluate LGI without the need of a blood sample. 

In our study, PWS adults showed smaller PhA and higher hs-CRP levels compared with control group matched for gender, age, and BMI. It is known that PhA is larger in males than in females [36]; however, while we also confirm this difference in our control group, there were no gender differences in the PhA in PWS adults. In addition, in agreement with previous evidence [11,18,37,38,39,40,41], both PhA and hs-CRP levels were negatively and positively associated with BMI and waist circumference, respectively. Of interest, the most important result of the present study is the negative correlation between PhA and hs-CRP levels, independently of gender, BMI, and waist circumference. Finally, based on the ROC curve analysis, the most sensitive and specific cut-off for the PhA to predict the highest hs-CRP levels was ≤4.8°. To the best of our knowledge, to date, this is the first study validating PhA as marker of inflammation in PWS adults.

PhA, obtained from BIA, has been used as a marker of nutritional status and as an indicator of cell membrane function in several populations [10]. In particular, smaller PhA is considered a marker for decreased cell membrane integrity or related to cell death [16], whereas larger PhA suggests a large number of intact cell membranes [10,42]. The tissue injury, and consequently, the cell membrane integrity disarrangement, is a well-known condition related to the inflammatory status [43]. Evidence in different clinical inflammatory conditions, including polycystic ovary syndrome [44], psoriasis [45,46], hidradenitis suppurativa [27], and obesity [19], showed a strong association between PhA and inflammatory markers, including hs-CRP levels [47,48]. The alterations of PhA also depend on age and gender, in particular, PhA is higher in males than females and decreases with aging [42]. Our data demonstrated that, unlike in the healthy subjects [11,36], in our group of PWS adults there was no evidence of gender-related or age-related differences in PhA. In fact, different PhA values in males and females are expected considering the different body cell mass and muscle mass [49,50]. This led us to speculate that LGI characterizing obesity and PWS adults could likely hinder the effects of gender and age on PhA in PWS adults.

Of interest, both PhA and inflammatory biomarker hs-CRP levels are related to body composition [11,12,13], especially FM [51,52,53]. 

Serum hs-CRP levels are elevated in individuals with overweight and obesity, especially visceral obesity, which is viewed as an LGI disease [54,55,56]. In particular, hs-CRP is one of the most important inflammatory markers and it is used as a common marker of obesity-related LGI [57] and is an independent cardiovascular risk predictor due to its direct involvement in the different phases of atheroma plaque formation [58]. Of interest, visceral adipose tissue seems to be the greatest target for leukocyte infiltration and also a source of inflammatory cytokines such as CRP levels [59,60]. In particular, CRP stimulates the production of pro-apoptotic cytokines including interleukin-1β (IL-1β), tumor necrosis factor-α (TNFα), and reactive oxygen species [61,62] and inflammatory mediators via the activation of Fc-γ receptors [63]. Nevertheless, our results reported that independently of BMI and waist circumference, the relationships between PhA and hs-CRP levels persist significantly, suggesting that PhA could be a relevant predictor of this marker in PWS adult. Nevertheless, this result needs to be confirmed in larger and prospective clinical trials. 

Additionally, the negative association between PhA and inflammation state in PWS adult could be an indirect effect of oxidative stress. In fact, high hs-CRP levels are associated to increased free radicals [64], which can cause cellular damage [65], and consequently could decrease PhA values. Although in our study we have not evaluated specific oxidative stress markers, and this conclusion remains only speculative. Therefore, our data suggest that PhA values were negatively associated with a pro-inflammatory state and that PhA may be a useful tool for indirectly evaluating it, independently of the of BMI and waist circumference.

In our results, we also found a correlation between PhA and hs-CRP levels with ECW/ICW ratio, regardless of gender, BMI, and waist circumference. 

PhA is negatively correlated to the ECW/ICW in healthy adults [13]. In fact, an increased ECW/ICW due to a rapid and early transfer of fluids from the ICW to the ECW compartment along with cell mass reductions is associated with smaller PhA. Thus, the inverse correlation reported between PhA and hs-CRP levels can be at least partly interpreted considering that the inflammatory response is the first defense against chemical and biological damage and for repair of tissue damage, so an exaggerated, disproportionate, or prolonged inflammatory state can determine tissue damage [66]. This LGI is closely linked to the development of several metabolic and cardiovascular diseases, also in PWS adults [67,68,69]. In our results, we reported that the hs-CRP levels were higher in PWS adults than in the control group, and this is in agreement with other studies performed in PWS adults [7,8,70,71]. Based on these results, we speculate that the PWS adults with the highest values of hs-CRP levels as inflammatory marker were those with lower cellular health, expressed as a smaller PhA. Reinforcing this result, we performed complementary analyzes to explore the association between ECW/ICW ratio and hs-CRP levels, as inflammatory biomarker. We found a negative correlation between ECW/ICW ratio with hs-CRP levels, regardless of gender, BMI, and waist circumference. The tight relation between ECW volume expansion and LGI [72,73,74], which involves a lower PhA, may partially determine the negative association of PhA with hs-CRP levels and ECW/ICW ratio. However, several studies have been carried out to date to elucidate the role of PhA as a prognostic and nutritional tool, and also as an indicator of health status in different clinical conditions [10]. Therefore, although our results can not demonstrate a causal link, they suggest that PhA could be a marker of LGI in PWS adults.

Some limitations should be illustrated in the present study. First, being a cross-sectional study, the cause–effect association between PhA and LGI can not be determined. 

Second, only one inflammatory marker, hs-CRP levels, was evaluated. Although hs-CRP is reported to be the most reliable unspecific biomarker of inflammatory state, we did not analyze other pro-inflammatory inflammatory markers such as TNF-α, which seems to be more associated with cell plasma membrane instability [75]. However, we point out that this was the first study reporting in PWS adults the relationship between PhA values and inflammation assessed by plasma hs-CRP levels, independently of BMI and waist circumference.

Third, the sample size was relatively small, and this is because PWS is considered a rare disease. Nevertheless, we have estimated the sample size using 95% statistical power, calculated by the differences of means of PhA in PWS adults and controls. However, in order to prevent any drop out, we enrolled double the number of patients required by sample size calculations for each group (15 patients for each group), matching controls for gender, age, and BMI. Furthermore, being a monocentric study, patients and controls shared the same geographical area with the same effect of latitude and, likely, similar food availability and dietary consumption patterns, which allowed us to improve the homogeneity of the study sample. 

Fourth, the proposed cut-off point of PhA for identifying the highest hs-CRP levels should be used with caution until an appropriate cross-validation is performed and the results of clinical trials in larger population samples are made available.

A strong point of our research is the significant association between PhA with hs-CRP levels after adjusting for relevant covariates, in particular, gender, BMI, and waist circumference, considering the power influence of these factors on PhA [11,76]. In addition, only the very same expert nutritionist performed and interpreted anthropometric measurements and BIA with the aim of minimizing inter-operator variability.

## 5. Conclusions

In summary, in the present study, we reported for the first time (i) smaller PhAs and higher ECW/ICW ratio as an expression of increased LGI in PWS adults compared with control group matched for gender, age, and BMI; (ii) in PWS adults, there were no age and sex differences in the PhA as was observed in the control group, thus leading us to speculate that the generalized inflammatory state characterizing obesity and PWS adults could likely hamper the effects of gender and age on PhA in PWS adults; (iii) a direct association between PhA and hs-CRP levels, independently of gender, BMI, waist circumference. As possible translational applications, these findings suggest that the identification of a specific cut-off value for PhA might help in identifying PWS adults at high risk of LGI who could benefit from a more detailed clinical evaluation. Since PWS adults have poor compliance due to their psychiatric disorders and most of the time is difficult to collect blood sampling, making the biochemical diagnosis of LGI not always possible, PhA could be a potential useful noninvasive biomarker of LGI, meeting the unmet needs in this population. In conclusion, our findings highlight the importance of nutritional assessment by a qualified nutritionist in adults with PWS, not only for providing nutritional advice but also to screen for LGI and to identify subjects in which to perform further biochemical assessments.

## Figures and Tables

**Table 1:** Weight and body composition parameters of the study population assessed by bioelectrical impedance analysis in PWS adults and control group.

Parameters	PWS Adults *n*= 15	Control Group *n* = 15	* *p*-Value
Weight (kg)	104.4 ± 34.4	120.5 ± 26.3	0.12
R (Ω)	445.6 ± 63.7	466.3 ± 53.3	0.28
Xc (Ω)	35.1 ± 9.4	45.8 ± 4.5	**0.001**
PhA (°)	4.5 ± 0.8	5.6 ± 0.3	**<0.001**
TBW (Lt)	42.7 ± 12.2	43.6 ± 4.7	0.79
ICW (Lt)	19.3 ± 5.7	22.9 ± 2.9	0.06
ECW (Lt)	23.4 ± 7.4	20.7 ± 1.8	0.18
ECW/ICW ratio	1.2 ± 0.3	0.9 ± 0.1	**<0.001**
FM (Kg)	49.9 ± 24.3	61.6 ± 22.6	0.10
FFM (Kg)	54.5 ± 13.7	58.9 ± 5.8	0.29

Significant higher values of ECW/ICW ratio were found in PWS adults compared with controls (*p* < 0.001), while lower values of Xc, and PhA, were found in PWS adults compared with control group. Results are expressed as mean ± SD. Age, weight, waist circumference, R, Xc, PhA, TBW, ICW, ECW, and FM were logarithmically normalized and transformed and back-transformed for presentation in the table. Differences between groups were analyzed by paired Student’s *t* test. * A *p-*value in bold type denotes a significant difference (*p* < 0.05). PWS, Prader–Willi syndrome; R, resistance; Xc, reactance; PhA, phase angle; TBW, total body water; ICW, intracellular water; ECW, extracellular water; FM, fat mass; FFM, fat-free mass.

**Table 2:** Correlations, simple and after adjustment for gender, BMI, and waist circumference, among PhA and hs-CRP levels with age and anthropometric measurements in PWS adults.

Parameters	PhA (°) *n* = 15				hs-CRP Levels (ng/mL) *n* = 15			
Simple Correlations		Adjusted for Gender, BMI, Waist Circumference		Simple Correlations		Adjusted for Gender, BMI, Waist Circumference		
	r	* *p*-Value	r	* *p*-Value	r	* *p*-Value	r	* *p*-Value
Age (years)	0.03	0.92	0.18	0.58	0.21	0.46	0.18	0.59
BMI (kg/m^2^)	−0.56	**0.03**	-	-	0.64	**0.01**	-	-
Waist circumference (cm)	−0.51	**0.04**	-	-	0.74	**0.002**	-	-
ICW (Lt)	0.17	0.54	0.88	**<0.001**	0.33	0.23	−0.77	**0.004**
ECW (Lt)	−0.56	**0.03**	−0.75	**0.01**	0.77	**0.001**	0.28	0.38
ECW/ICW ratio	−0.99	**<0.001**	−0.99	**<0.001**	0.67	**0.01**	0.68	**0.01**
FM (Kg)	−0.60	**0.02**	−0.18	0.57	0.66	**0.01**	−0.01	0.96

PhA and hs-CRP levels were correlated with anthropometric measurements and most BIA parameters. After adjustment for covariates, significant correlations were found between PhA and hs-CRP levels with ICW, and ECW/ICW ratio. Age, weight, waist circumference, PhA, ICW, ECW, and FM were logarithmically normalized and transformed and back-transformed for presentation in the table. Correlations among variables were performed using Pearson *r* correlation coefficients. * A *p-*value in bold type denotes a significant difference (*p* < 0.05). PhA, phase angle; hs-CRP, high-sensitivity C-reactive protein; BMI, body mass index; ICW, intracellular water; ECW, extracellular water; FM, fat mass.

**Table 3:** Multiple regression analysis models (stepwise method) with the PhA as dependent variable to estimate the predictive value of hs-CRP levels, BMI, waist circumference, and BIA parameters in PWS adults.

Parameters	Multiple Regression Analysis			
	R^2^	β	t	* *p*-Value
**ECW/ICW ratio**	0.98	−0.94	−26.8	**<0.001**
**hs-CRP levels**	0.99	0.08	−2.5	**0.02**
Excluded variables: BMI, waist circumference, and ECW.				

* A *p-*value in bold type denotes a significant difference (*p* < 0.05).

## Supplementary Materials

The following are available online at https://www.mdpi.com/2072-6643/12/7/2065/s1, Table S1: Correlations simple and after adjusted for gender, BMI, and waist circumference among PhA and hs-CRP levels with age and anthropometric measurements in control group.

## Abbreviations

***PWS:*** Prader–Willi syndrome; ***hs-CRP***, high-sensitivity C-reactive protein; ***LGI,*** chronic low-grade systemic inflammation; ***PhA,*** phase angle; ***BIA,*** bioelectrical impedance analysis; ***Xc,*** reactance; ***R,*** resistance; ***ICW,*** intracellular water; ***ECW,*** extracellular water; ***SD,*** standard deviations; ***TBW,*** total body water; ***FM,*** fat mass; ***FFM,*** fat-free mass; ***ROC,*** receiver operator characteristic; ***AUC,*** area under the curve; ***CI,*** confidence interval.

## References

[1] Cassidy S.B., Schwartz S., Miller J.L., Driscoll D.J. (2012). Prader-Willi syndrome. Genet. Med..

[2] Goldstone A.P., Holland A.J., Hauffa B.P., Hokken-Koelega A.C., Tauber M. (2008). Recommendations for the diagnosis and management of Prader-Willi syndrome. J. Clin. Endocrinol. Metab..

[3] Whittington J., Holland A., Webb T., Butler J., Clarke D., Boer H. (2004). Cognitive abilities and genotype in a population-based sample of people with Prader-Willi syndrome. J. Intellect. Disabil. Res..

[4] Alfaro D.L.P., Lemoine P., Ehlinger V., Molinas C., Diene G., Valette M., Pinto G., Coupaye M., Poitou-Bernert C., Thuilleaux D. (2019). Causes of death in Prader-Willi syndrome: Lessons from 11 years’ experience of a national reference center. Orphanet J. Rare Dis..

[5] Chawla A., Nguyen K.D., Goh Y.P.S. (2011). Macrophage-Mediated inflammation in metabolic disease. Nat. Rev. Immunol..

[6] Zatterale F., Longo M., Naderi J., Raciti G.A., Desiderio A., Miele C., Beguinot F. (2020). Chronic Adipose Tissue Inflammation Linking Obesity to Insulin Resistance and Type 2 Diabetes. Front. Physiol..

[7] Butler M.G., Bittel D.C., Kibiryeva N., Garg U. (2006). C-Reactive protein levels in subjects with Prader-Willi syndrome and obesity. Genet. Med..

[8] Caixàs A., Giménez-Palop O., Broch M., Vilardell C., Megía A., Simón I., Giménez-Pérez G., Mauricio D., Vendrell J., Richart C. (2008). Adult subjects with Prader-Willi syndrome show more low-grade systemic inflammation than matched obese subjects. J. Endocrinol. Invest..

[9] Höybye C. (2006). Inflammatory markers in adults with prader-willi syndrome before and during 12 months growth hormone treatment. Horm. Res..

[10] Barbosa-Silva M.C.G., Barros A.J.D. (2005). Bioelectrical impedance analysis in clinical practice: A new perspective on its use beyond body composition equations. Curr. Opin. Clin. Nutr. Metab. Care.

[11] Norman K., Stobäus N., Pirlich M., Bosy-Westphal A. (2012). Bioelectrical phase angle and impedance vector analysis - Clinical relevance and applicability of impedance parameters. Clin. Nutr..

[12] Stobäus N., Pirlich M., Valentini L., Schulzke J.D., Norman K. (2012). Determinants of bioelectrical phase angle in disease. Br. J. Nutr..

[13] Gonzalez M.C., Barbosa-Silva T.G., Bielemann R.M., Gallagher D., Heymsfield S.B. (2016). Phase angle and its determinants in healthy subjects: Influence of body composition. Am. J. Clin. Nutr..

[14] Kyle U.G., Bosaeus I., De Lorenzo A.D., Deurenberg P., Elia M., Gómez J.M., Heitmann B.L., Kent-Smith L., Melchior J.C., Pirlich M. (2004). Bioelectrical impedance analysis–Part II: Utilization in clinical practice. Clin. Nutr..

[15] Pham-Huy L.A., He H., Pham-Huy C. (2008). Free radicals, antioxidants in disease and health. Int. J. Biomed. Sci..

[16] Selberg O., Selberg D. (2002). Norms and correlates of bioimpedance phase angle in healthy human subjects, hospitalized patients, and patients with liver cirrhosis. Eur. J. Appl. Physiol..

[17] Norman K., Stobäus N., Zocher D., Bosy-Westphal A., Szramek A., Scheufele R., Smoliner C., Pirlich M. (2010). Cutoff percentiles of bioelectrical phase angle predict functionality, quality of life, and mortality in patients with cancer. Am. J. Clin. Nutr..

[18] De Luis D.A., Aller R., Romero E., Dueñas A., Perez Castrillon J.L. (2010). Relation of phase angle tertiles with blood adipocytokines levels, insulin resistance and cardiovascular risk factors in obese women patients. Eur. Rev. Med. Pharmacol. Sci..

[19] Barrea L., Muscogiuri G., Laudisio D., Di Somma C., Salzano C., Pugliese G., de Alteriis G., Colao A., Savastano S. (2019). Phase angle: A possible biomarker to quantify inflammation in subjects with obesity and 25(OH)D deficiency. Nutrients.

[20] Barrea L., Muscogiuri G., Pugliese G., Aprano S., de Alteriis G., Di Somma C., Colao A., Savastano S. (2020). The Sun’s Vitamin in Adult Patients Affected by Prader–Willi Syndrome. Nutrients.

[21] ClinCalc.com Sample Size Calculator. https://clincalc.com/stats/samplesize.aspx.

[22] Barrea L., Annunziata G., Muscogiuri G., Laudisio D., Di Somma C., Maisto M., Tenore G.C., Colao A., Savastano S. (2019). Trimethylamine N-oxide, Mediterranean diet, and nutrition in healthy, normal-weight adults: also a matter of sex?. Nutrition.

[23] Barrea L., Muscogiuri G., Di Somma C., Tramontano G., De Luca V., Illario M., Colao A., Savastano S. (2018). Association between Mediterranean diet and hand grip strength in older adult women. Clin. Nutr..

[24] Savastano S., Di Somma C., Colao A., Barrea L., Orio F., Finelli C., Pasanisi F., Contaldo F., Tarantino G. (2015). Preliminary data on the relationship between circulating levels of Sirtuin 4, anthropometric and metabolic parameters in obese subjects according to growth hormone/insulin-like growth factor-1 status. Growth Horm. IGF Res..

[25] WHO World Health Organization. http://www.euro.who.int/en/health-topics/disease-prevention/nutrition/a-healthy-lifestyle/body-mass-index-bmi.

[26] Nishida C., Ko G.T., Kumanyika S. (2010). Body fat distribution and noncommunicable diseases in populations: Overview of the 2008 WHO Expert Consultation on Waist Circumference and Waist-Hip Ratio. Eur. J. Clin. Nutr..

[27] Barrea L., Fabbrocini G., Annunziata G., Muscogiuri G., Donnarumma M., Marasca C., Colao A., Savastano S. (2019). Role of nutrition and adherence to the mediterranean diet in the multidisciplinary approach of hidradenitis suppurativa: Evaluation of nutritional status and its association with severity of disease. Nutrients.

[28] Barrea L., Altieri B., Muscogiuri G., Laudisio D., Annunziata G., Colao A., Faggiano A., Savastano S. (2018). Impact of nutritional status on gastroenteropancreatic neuroendocrine tumors (GEP-NET) aggressiveness. Nutrients.

[29] Muscogiuri G., Barrea L., Di Somma C., Laudisio D., Salzano C., Pugliese G., de Alteriis G., Colao A., Savastano S. (2019). Sex differences of vitamin D status across BMI classes: An observational prospective cohort study. Nutrients.

[30] Kushner R.F. (1992). Bioelectrical impedance analysis: A review of principles and applications. J. Am. Coll. Nutr..

[31] Yanovski S.Z., Hubbard V.S., Heymsfield S.B., Lukaski H.C. (1996). Bioelectrical impedance analysis in body composition measurement: National institutes of health technology assessment conference statement. Am. J. Clin. Nutr..

[32] Bedogni G., Grugni G., Tringali G., Agosti F., Sartorio A. (2015). Assessment of fat-free mass from bioelectrical impedance analysis in obese women with Prader-Willi syndrome. Ann. Hum. Biol..

[33] Bedogni G., Grugni G., Tringali G., Tamini S., Marzullo P., Sartorio A. (2019). Assessment of fat-free mass from bioelectrical impedance analysis in men and women with Prader-Willi syndrome: Cross-Sectional study. Int. J. Food Sci. Nutr..

[34] Gray D.S., Bray G.A., Gemayel N., Kaplan K. (1989). Effect of obesity on bioelectrical impedance. Am. J. Clin. Nutr..

[35] Lazzer S., Grugni G., Tringali G., Sartorio A. (2016). Prediction of basal metabolic rate in patients with Prader-Willi syndrome. Eur. J. Clin. Nutr..

[36] Barbosa-Silva M.C.G., Barros A.J.D., Wang J., Heymsfield S.B., Pierson R.N. (2005). Bioelectrical impedance analysis: Population reference values for phase angle by age and sex. Am. J. Clin. Nutr..

[37] Choi J., Joseph L., Pilote L. (2013). Obesity and C-reactive protein in various populations: A systematic review and meta-analysis. Obes. Rev..

[38] Kao T.W., Lu I.S., Liao K.C., Lai H.Y., Loh C.H., Kuo H.K. (2009). Associations between body mass index and serum levels of C-reactive protein. S. Afr. Med. J..

[39] Park H.S., Park J.Y., Yu R. (2005). Relationship of obesity and visceral adiposity with serum concentrations of CRP, TNF-α and IL-6. Diabetes Res. Clin. Pract..

[40] Nakamura H., Ito H., Egami Y., Kaji Y., Maruyama T., Koike G., Jingu S., Harada M. (2008). Waist circumference is the main determinant of elevated C-reactive protein in metabolic syndrome. Diabetes Res. Clin. Pract..

[41] Anja B.W., Danielzik S., Dörhöfer R.P., Later W., Wiese S., Müller M.J. (2006). Phase angle from bioelectrical impedance analysis: Population reference values by age, sex, and body mass index. J. Parenter. Enter. Nutr..

[42] Buffa R., Floris G., Marini E. (2003). Migration of the bioelectrical impedance vector in healthy elderly subjects. Nutrition.

[43] Lowe G.D.O., Fowkes F.G.R., Dawes J., Donnan P.T., Lennie S.E., Housley E. (1993). Blood viscosity, fibrinogen, and activation of coagulation and leukocytes in peripheral arterial disease and the normal population in the Edinburgh artery study. Circulation.

[44] Barrea L., Arnone A., Annunziata G., Muscogiuri G., Laudisio D., Salzano C., Pugliese G., Colao A., Savastano S. (2019). Adherence to the mediterranean diet, dietary patterns and body composition in women with polycystic ovary syndrome (PCOS). Nutrients.

[45] Barrea L., Macchia P.E., Di Somma C., Napolitano M., Balato A., Falco A., Savanelli M.C., Balato N., Colao A., Savastano S. (2016). Bioelectrical phase angle and psoriasis: A novel association with psoriasis severity, quality of life and metabolic syndrome. J. Transl. Med..

[46] Barrea L., Nappi F., Di Somma C., Savanelli M.C., Falco A., Balato A., Balato N., Savastano S. (2016). Environmental risk factors in psoriasis: The point of view of the nutritionist. Int. J. Environ. Res. Public Health.

[47] Demirci M.S., Demirci C., Ozdogan O., Kircelli F., Akcicek F., Basci A., Ok E., Ozkahya M. (2011). Relations between malnutritioninflammationatherosclerosis and volume status. The usefulness of bioimpedance analysis in peritoneal dialysis patients. Nephrol. Dial. Transplant..

[48] Tomeleri C.M., Cavaglieri C.R., de Souza M.F., Cavalcante E.F., Antunes M., Nabbuco H.C.G., Venturini D., Barbosa D.S., Silva A.M., Cyrino E.S. (2018). Phase angle is related with inflammatory and oxidative stress biomarkers in older women. Exp. Gerontol..

[49] Xu Y., Xie X., Duan Y., Wang L., Cheng Z., Cheng J. (2016). A review of impedance measurements of whole cells. Biosens. Bioelectron..

[50] Marini E., Buffa R., Saragat B., Coin A., Toffanello E.D., Berton L., Manzato E., Sergi G. (2012). The potential of classic and specific bioelectrical impedance vector analysis for the assessment of sarcopenia and sarcopenic obesity. Clin. Interv. Aging.

[51] Li F., Li Y., Duan Y., Hu C.A.A., Tang Y., Yin Y. (2017). Myokines and adipokines: Involvement in the crosstalk between skeletal muscle and adipose tissue. Cytokine Growth Factor Rev..

[52] Tilg H., Moschen A.R. (2006). Adipocytokines: Mediators linking adipose tissue, inflammation and immunity. Nat. Rev. Immunol..

[53] Berg A.H., Scherer P.E. (2005). Adipose tissue, inflammation, and cardiovascular disease. Circ. Res..

[54] Fantuzzi G. (2005). Adipose tissue, adipokines, and inflammation. J. Allergy Clin. Immunol..

[55] Greenberg A.S., Obin M.S. (2006). Obesity and the role of adipose tissue in inflammation and metabolism. Am. J. Clin. Nutr..

[56] Trayhurn P., Wang B., Wood I.S. (2008). Hypoxia in adipose tissue: A basis for the dysregulation of tissue function in obesity?. Br. J. Nutr..

[57] Bisoendial R.J., Birjmohun R.S., Akdim F., van ’t Veer C., Spek C.A., Hartman D., de Groot E.R., Bankaitis-Davis D.M., Kastelein J.J.P., Stroes E.S.G. (2009). C-Reactive Protein Elicits White Blood Cell Activation in Humans. Am. J. Med..

[58] Avan A., Tavakoly Sany S.B., Ghayour-Mobarhan M., Rahimi H.R., Tajfard M., Ferns G. (2018). Serum C-reactive protein in the prediction of cardiovascular diseases: Overview of the latest clinical studies and public health practice. J. Cell. Physiol..

[59] Ragino Y.I., Stakhneva E.M., Polonskaya Y.V., Kashtanova E.V. (2020). The role of secretory activity molecules of visceral adipocytes in abdominal obesity in the development of cardiovascular disease: A review. Biomolecules.

[60] Tchernof A., Després J.P. (2013). Pathophysiology of human visceral obesity: An update. Physiol. Rev..

[61] Kobayashi S., Inoue N., Ohashi Y., Terashima M., Matsui K., Mori T., Fujita H., Awano K., Kobayashi K., Azumi H. (2003). Interaction of oxidative stress and inflammatory response in coronary plaque instability: Important role of C-reactive protein. Arterioscler. Thrombosis Vasc. Biol..

[62] Ryu J., Lee C.W., Shin J.A., Park C.S., Kim J.J., Park S.J., Han K.H. (2007). FcγRIIa mediates C-reactive protein-induced inflammatory responses of human vascular smooth muscle cells by activating NADPH oxidase 4. Cardiovasc. Res..

[63] Sproston N.R., Ashworth J.J. (2018). Role of C-reactive protein at sites of inflammation and infection. Front. Immunol..

[64] Kotani K., Sakane N. (2012). C-reactive protein and reactive oxygen metabolites in subjects with metabolic syndrome. J. Int. Med. Res..

[65] Janssen Y.M., Van Houten B., Borm P.J., Mossman B.T. (1993). Cell and tissue responses to oxidative damage. Lab. Invest. A J. Tech. Methods Pathol..

[66] Chen L., Deng H., Cui H., Fang J., Zuo Z., Deng J., Li Y., Wang X., Zhao L. (2018). Inflammatory responses and inflammation-associated diseases in organs. Oncotarget.

[67] Muscogiuri G., Formoso G., Pugliese G., Ruggeri R.M., Scarano E., Colao A. (2019). Prader- Willi syndrome: An uptodate on endocrine and metabolic complications. Rev. Endocr. Metab. Disord..

[68] Crinò A., Fintini D., Bocchini S., Grugni G. (2018). Obesity management in Prader–Willi syndrome: Current perspectives. Diabetes Metab. Syndr. Obes. Targets Ther..

[69] Olsson L.M., Poitou C., Tremaroli V., Coupaye M., Aron-Wisnewsky J., Bäckhed F., Clément K., Caesar R. (2019). Gut microbiota of obese subjects with Prader-Willi syndrome is linked to metabolic health. Gut.

[70] van Nieuwpoort I.C., Twisk J.W.R., Curfs L.M.G., Lips P., Drent M.L. (2018). Body composition, adipokines, bone mineral density and bone remodeling markers in relation to IGF-1 levels in adults with Prader-Willi syndrome. Int. J. Pediatr. Endocrinol..

[71] Faienza M.F., Ventura A., Lauciello R., Crinò A., Ragusa L., Cavallo L., Spera S., Grugni G. (2012). Analysis of endothelial protein c receptor gene and metabolic profile in prader-willi syndrome and obese subjects. Obesity.

[72] Ortega O., Gallar P., Muñoz M., Rodríguez I., Carreño A., Ortiz M., Molina A., Oliet A., Lozano L., Vigil A. (2004). Association between C-Reactive Protein Levels and N-Terminal Pro-B-Type Natriuretic Peptide in Pre-Dialysis Patients. Nephron Clin. Pract..

[73] Gonçalves S., Pecoits-Filho R., Perreto S., Barberato S.H., Stinghen A.E.M., Lima E.G.A., Fuerbringer R., Sauthier S.M., Riella M.C. (2006). Associations between renal function, volume status and endotoxaemia in chronic kidney disease patients. Nephrol. Dial. Transplant..

[74] Sprague A.H., Khalil R.A. (2009). Inflammatory cytokines in vascular dysfunction and vascular disease. Biochem. Pharmacol..

[75] Cai Z., Jitkaew S., Zhao J., Chiang H.C., Choksi S., Liu J., Ward Y., Wu L.G., Liu Z.G. (2014). Plasma membrane translocation of trimerized MLKL protein is required for TNF-induced necroptosis. Nat. Cell Biol..

[76] Kyle U.G., Soundar E.P., Genton L., Pichard C. (2012). Can phase angle determined by bioelectrical impedance analysis assess nutritional risk? A comparison between healthy and hospitalized subjects. Clin. Nutr..

