# Supplementary material for: Phase Angle: Could Be an Easy Tool to Detect Low-Grade Systemic Inflammation in Adults Affected by Prader–Willi Syndrome?
Source: Nutrients. 2020 Jul 11;12(7):2065. doi: 10.3390/nu12072065 (PMC7400955; doi:10.3390/nu12072065)
Supplement: Supplementary file 1 [file nutrients-12-02065-s001.zip › nutrients-848712-supplementary/Supplementary Materials.docx]

*Supplementary Materials*

**Material and Methods**

*Anthropometric measurements*

Parameters were measurements after an overnight fast in the morning between 8 and 10 am. The degree of obesity was established according to World Health Organization’s criteria: Grade I obesity (body mass index: 30.0–34.9 kg/m^2^), grade II obesity (body mass index: 35.0–39.9 kg/m^2^), grade III obesity (body mass index ≥40.0 kg/m^2^). A single nutritionist measured waist circumference with a non-stretchable tailor measuring tape was placed around the bare abdomen just above the hip bone and parallel to the floor. Patients and controls were asked to exhale, and measurement was taken to the nearest centimeter at the midpoint between the bottom of the rib cage and above the top of the iliac crest during minimal respiration.

*Body composition*

All BIA-measurements were performed under strictly standardized conditions by a single nutritionist, using the same device in order to avoid interobserver and interdevice variability. The instrument was routinely checked with resistors and capacitors of known values. Reliability for within-day and between-day measurements by the same observer were <1.9% for resistance, <2.1% for reactance, and <2.8% for resistance, <2.5% for reactance, respectively. The coefficients of variation of repeated measurements of resistance and reactance at 50 kHz was assessed in 6 individuals (3 males and 3 females) by the same observer: coefficients of variation were 1.6% for resistance and 1.4% for reactance. The phase angle was derived from conditions under 50 kHz according to the following formula: phase angle (°, degrees) = arctangent reactance / resistance ((reactance / resistance) × (180/π)).
