# Supplementary material for: Phase Angle: Could Be an Easy Tool to Detect Low-Grade Systemic Inflammation in Adults Affected by Prader–Willi Syndrome?
Source: Nutrients. 2020 Jul 11;12(7):2065. doi: 10.3390/nu12072065 (PMC7400955; doi:10.3390/nu12072065)
Supplement: Supplementary file 1 [file nutrients-12-02065-s001.zip › nutrients-848712-supplementary/Supplementary tables.docx]

| **Parameters.** | PhA (°)  n=15 | | | |  | hs-CRP levels (ng/mL)  n=15 | | |
| --- | --- | --- | --- | --- | --- | --- | --- | --- |
|  | **Simple**  **correlations** | | **Adjusted**  **for gender, BMI,**  **waist circumference** | | **Simple correlations** | | **Adjusted**  **for gender**, **BMI, waist circumference** | |
|  | **r** | ****p*-value** | **r** | ****p*-value** | **r** | ****p*-value** | **r** | ****p*-value** |
| Age (years) | -0.06 | 0.82 | 0.91 | 0.77 | -0.01 | 0.98 | 0.03 | 0.96 |
| BMI (kg/m^2^) | 0.04 | 0.89 | - | - | 0.14 | 0.62 | - | - |
| Waist circumference (cm) | -0.25 | 0.37 | - | - | -0.39 | 0.15 | - | - |
| ICW (Lt) | 0.78 | **0.01** | 0.83 | **<0.001** | -0.62 | **0.01** | 0.73 | **0.01** |
| ECW (Lt) | -0.43 | 0.12 | 0.45 | 0.13 | 0.48 | 0.06 | -0.69 | **0.01** |
| ECW/ICW ratio | -0.99 | **<0.001** | -0.99 | **<0.001** | 0.55 | **0.04** | -0.49 | 0.09 |
| FM (Kg) | 0.11 | 0.70 | -0.09 | 0.76 | 0.17 | 0.55 | -0.27 | 0.37 |

**Supplementary Table 1. Correlations simple and after adjusted for gender, BMI, and waist circumference among PhA and hs-CRP levels with age and anthropometric measurements in control group.**

After adjustment for covariates, significant correlation were found between PhA and hs-CRP levels with ICW, and ECW/ICW ratio. Age, weight, waist circumference, PhA, ICW, ECW, and FM, were logarithmically normalized and transformed and back-transformed for presentation in table. Correlations among variables were performed using Pearson *r* correlation coefficients. *A *p* value in bold type denotes a significant difference (*p*<0.05).

**PhA,** Phase Angle; **hs-CRP,** High Sensitivity C-reactive Protein; **BMI,** Body Mass Index; **R,** Resistance; **Xc,** Reactance; **TBW,** Total Body Water; **ICW,** Intra-cellular Water; **ECW,** Extra-cellular Water; **FM,** Fat Mass; **FFM,** Free Fat Mass.
